# PE-Net: a parallel framework for 3D inferior mesenteric artery segmentation

**DOI:** 10.3389/fphys.2023.1308987

**Published:** 2023-12-11

**Authors:** Kun Zhang, Peixia Xu, Meirong Wang, Pengcheng Lin, Danny Crookes, Bosheng He, Liang Hua

**Affiliations:** ^1^ School of Electrical Engineering, Nantong University, Nantong, Jiangsu, China; ^2^ Nantong Key Laboratory of Intelligent Control and Intelligent Computing, Nantong Institute of Technology, Nantong, Jiangsu, China; ^3^ Nantong Key Laboratory of Intelligent Medicine Innovation and Transformation, Affiliated Hospital 2 of Nantong University, Nantong, Jiangsu, China; ^4^ Department of Radiology, Affiliated Hospital 2 of Nantong University, Nantong, Jiangsu, China; ^5^ School of Electronics, Electrical Engineering and Computer Science, Queen’s University Belfast, Belfast, United Kingdom; ^6^ Clinical Medicine Research Center, Affiliated Hospital 2 of Nantong University, Nantong, Jiangsu, China

**Keywords:** vessel volume, transformer, axial attention, edge feature, parallel encoding

## Abstract

The structural morphology of mesenteric artery vessels is of significant importance for the diagnosis and treatment of colorectal cancer. However, developing automated vessel segmentation methods for this purpose remains challenging. Existing convolution-based segmentation methods have limitations in capturing long-range dependencies, while transformer-based models require large datasets, making them less suitable for tasks with limited training samples. Moreover, over-segmentation, mis-segmentation, and vessel discontinuity are common challenges in vessel segmentation tasks. To address these issues, we propose a parallel encoding architecture that combines transformers and convolutions to retain the advantages of both approaches. The model effectively learns position deviations and enhances robustness for small-scale datasets. Additionally, we introduce a vessel edge capture module to improve vessel continuity and topology. Extensive experimental results demonstrate the improved performance of our model, with Dice Similarity Coefficient and Average Hausdorff Distance scores of 81.64% and 7.7428, respectively.

## 1 Introduction

According to the global cancer burden data released by the International Agency for Research on Cancer of the World Health Organization in 2020, colorectal cancer has become the third most common cancer and the second most deadly cancer worldwide, following only lung cancer and liver cancer, with an increasing incidence among young and middle-aged populations (Xi and Xu, 2021). Surgery is the primary approach for curative treatment of colorectal cancer, involving tumor resection, ligation of local blood vessels, and lymph node dissection. The inferior mesenteric artery (IMA) is a key site for lymph node metastasis and the target vessel for ligation (Yada et al., 1997). Preoperative knowing the arterial branching helps surgeons create surgical plans for the safe and effective ligation of arteries and lymph node clearing.

Over the years, convolutional neural networks (CNNs) have greatly contributed to the field of computer vision, owing to their excellent feature extraction and expression capabilities. They have been widely used for tasks such as classification, segmentation, object detection, and registration. In 2015, the success of the Unet (Ronneberger et al., 2015) established the important position of CNNs in medical image analysis, and many variants based on Unet have been subsequently proposed, which have achieved impressive results in 2D medical image analysis. In 2017, 3D Unet (Çiçek et al., 2016) was introduced for the processing of 3D medical images, which further propelled the development of CNNs in 3D medical imaging tasks. Many automatic segmentation algorithms have been proposed based on these models, involving organs (Garcia-Uceda Juarez et al., 2019; Chen et al., 2020), tissues (Chen et al., 2017), tumors (Feng et al., 2020; Liu et al., 2015), and many other targets. Given the remarkable performance of CNNs in pixel (voxel) segmentation tasks, it has also been widely used for vessel segmentation (Zhao et al., 2022; Pan et al., 2022; Li et al., 2022). However, for some small vessels, the segmented results are often not accurate enough.

Many attempts have been made to expand the receptive field of convolutional networks to capture more global information. Wu et al. (2019) proposed a dilated convolution which can expand the receptive field, and achieved excellent performance in multiple segmentation tasks. Zhao et al. (2017) designed a multi-scale feature pyramid to aggregate more global information. Peng et al. (2017) applied a large kernel to capture global relationships. Although these methods have improved the modeling of contextual relationships to some extent, these models are still limited by the restricted receptive field of convolutional architectures.Compared with convolutional networks, the transformer relaxes the local inductive bias, enhances the interaction between non-local regions, and allows for effective learning of long-range information. Given the outstanding performance of the transformer, many methods have attempted to introduce it into the field of medical image processing. Dosovitskiy et al. (2020) proposed Vision Transformer (ViT), which was the first attempt to use the transformer for vision tasks. Liu et al. (2021) proposed a hierarchical architecture that uses movable windows to allow attention to be local and across-window connections to improve computational efficiency, making it highly compatible with various visual tasks. Some recent methods have attempted to combine CNN and transformer to improve model performance (Chen et al., 2021; Xie et al., 2021; Wang et al., 2021). However, these networks still rely heavily on convolutional layers, and the transformer is only embedded as a separate module to compensate for the lack of long-range relationships in the features extracted by the convolution. Specifically, they are often arranged after the convolutional feature extraction module in each layer or part of the feature compact layer. When the feature is feeded into the transformer, it is usually a limited feature that has undergone convolutional operations. We believe that it is usually limited to compensate for global dependencies based on this foundation, and the performance potential of the transformer is not fully exploited.

Due to the lack of inductive bias of transformers for images, transformer-based models require training on large-scale datasets or extensive pre-training to perform effectively (Dosovitskiy et al., 2020). This poses a problem when using Transformers for small scale dataset, which is a common problem in medical imaging. To address this, we propose a Triple-Axial Gated Transformer (TAGT) that runs the transformer from three directions: height, width, and depth, greatly enhancing the sensitivity of positional information, making the model more versatile and not restricted by massive amounts of data.

**FIGURE 1:**
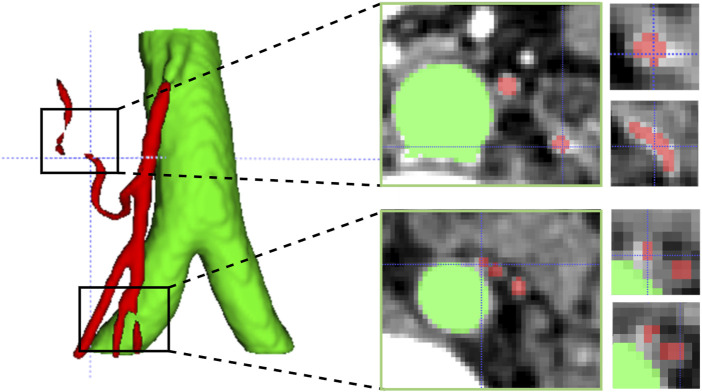
Challenges in segmenting tubular vessels. The first column highlights two instances of vascular rupture identified in the 3D predictive model. The second column displays cross-sectional slices corresponding to the aforementioned ruptures. The third column presents magnified images of two adjacent microvessels extracted from the vicinity of the corresponding slices.

In addition, vascular images often exhibit sparse, elongated tubular structures. Due to uneven noise, low contrast, and the complex topology of blood vessels, existing methods for vessel segmentation typically suffer from the following problems: over-segmentation or mis-segmentation, poor vascular continuity, and poor capturing of microvessels. Therefore, 3D elongated tubular vessel segmentation remains a topic worthy of joint research. We attribute the above problems to the insensitivity to vessel edge structures. In Figure 1, we show an example of the IMA vessel prediction results, and even an error of a few pixels can have a huge impact on the continuity of vessels. Inspired by self-attention, we designed a vessel edge-sensitive module that enhances the capturing ability of vessel edges by increasing the weight of edge voxels in the vessel volume image.

CNNs possess translational invariance but lack global feature comprehension. On the other hand, transformers excel at capturing global context, but their lack of translational invariance demands ample training data. Thus, their advantages complement each other. In existing architectural paradigms, some fusion architectures, predominantly relying on convolutional layers, tend to replace convolutions with transformers in a few compact layers. However, we posit that in limited convolutional features, leveraging transformers to capture global features may not fully exploit the advantages of transformers. In contrast to existing architectures, our approach involves constructing parallel branches for CNNs and transformers, aiming to maximize the utilization of their respective strengths.

In this paper, we explore a parallel encoding architecture that tightly integrates transformers with convolutional networks to address automatic segmentation of the inferior mesenteric artery in the abdomen. Our contributions can be summarized as follows:

1. We propose a parallel connection approach for integrating CNN and transformer, retaining the inductive bias of convolutions and the ability of transformers to model long-range dependencies. The architecture follows the classic encoder-decoder structure.
2. We extend the axial attention mechanism to the 3D domain, computing attention along the width, height, and depth directions. This efficient learning of positional information enhances the model’s ability to focus on fine details in small regions, addressing the issue of transformers struggling to learn image position encoding on small datasets and improving the model’s robustness for tasks with limited data.
3. We introduce a vessel edge feature capture (EFC) module which enhances the weight of vessel edge voxels to improve vessel boundary extraction and enhance vessel continuity, especially for capturing fine vessel boundaries.
4. We design a deep feature fusion block (FFB) to allocate weights between high-level features generated by the decoder and low-level features from skip connections. This selective feature fusion retains prominent features relevant to vessel structures.

### 1.1 Prior work and challenges

We built our Parallel Encoding Net (PE-Net) based on the latest successful foundations of convolutional neural networks and transformers. In this section, we briefly review the relevant methods and expand on two subfields: convolutional segmentation networks and semantic segmentation using transformers.

#### 1.1.1 Semantic segmentation using ConvsNet

CNNs have achieved tremendous success in various visual tasks such as segmentation, classification, registration, and object detection. Among them, the fully convolutional network (FCN) has become the mainstream network for semantic segmentation and has inspired many deeper and larger networks. In particular, the U-Net (Ronneberger et al., 2015) architecture, which uses a decoding-encoding structure with skip connections, has become the mainstream architecture for medical image segmentation. Many U-Net variants, such as U-Net++ (Zhou et al., 2018) and Res-UNet (Zhang et al., 2018), have further improved the performance of image segmentation. The W-UNET (Hong et al., 2019) improves segmentation performance by stacking multiple decoding-encoding modules, while the 3D Unet and V-Net (Milletari et al., 2016) further extend these high-quality features to the segmentation of 3D images. nnUnet (Isensee et al., 2021) is a significant breakthrough in the field of medical image segmentation using the U-Net architecture, which adapts training parameters to perform well in both 2D and 3D tasks and ranks first in the top ten tasks without changing the network structure.

Despite the success of these convolutional networks, the locality of the convolutional layers in CNNs limits their ability to learn distant spatial correlations. The convolution operation used in these models captures texture features by collecting local information from neighboring pixels. To aggregate feature filter responses at the global scale, many solutions have been proposed, which can be broadly classified into using dilated convolutions (Wu et al., 2019), increasing kernel size (Chen et al., 2017), adopting feature pyramid pooling (Chen et al., 2017; Zhao et al., 2017), and non-local operations (Wang et al., 2018). Although these methods have been shown to improve performance, such improvements are limited and cannot completely solve this problem.

#### 1.1.2 Semantic segmentation using transformer

In recent years, a large number of excellent transformer-based methods have emerged in the field of image processing. Transformer-based visual models can be further divided into two types: one is the method constructed mainly with convolutional layers, and the other is the method using transformer as the main architecture. TransUnet (Chen et al., 2021) is proposed as the first attempt to introduce the transformer into the field of medical image segmentation. The core idea is to embed transformer blocks between the CNN encoder and decoder to capture long-range dependencies. The idea of embedding several layers of transformers in a convolutional network has attracted a lot of followers. TransBTS (20) extends the transformer to 3D medical image processing tasks, modeling the remote dependencies in depth and spatial dimensions. Its structure is similar to TransUnet, placing transformers at the bottom of the U-shaped network. Wang et al. (2022) proposed a hybrid framework, using convolution as a shallow feature extractor in the first three layers and performing transformers in the last two layers. UTNET (Hatamizadeh et al., 2020) replaces a group of convolutions in the encoding and decoding layers of the U-shaped network with transformer blocks. Cotr (Xie et al., 2021) proposes a mixed architecture to efficiently bridge CNN and transformer and introduces a deformable attention mechanism to reduce the computational complexity for incorporating more transformer layers. However, these methods typically treat the transformer as a module embedded or replaced within a few layers of convolutional networks, without fully overcoming the inherent limitations of feature extraction with convolutions. This has prompted researchers to explore solutions based on the transformer architecture.

Some researchers lead the way in using transformer as a feature extractor. Zheng et al. (2021) deploy a pure transformer network to encode images as a series of patches, without using any convolutions or downsampling operations, and utilize each layer of the transformer for context modeling. Swin Transformer (Liu et al., 2021) is proposed to apply the inductive bias of CNN to the transformer architecture, allowing window movement and computing local attention in each small block window for global interaction. Zhou et al. (2021) integrated the advantages of the nnUnet architecture and employed Swin Transformer in both the encoder and decoder, achieving impressive performance. Nonetheless, as the transformer architecture relies on attention mechanisms to capture holistic information, its inherent lack of translational invariance often demands an extensive volume of training data or pre-trained models. This predicament renders it arduous to cater to the demands of small training datasets. Tragakis et al. (2023) introduced a novel fully convolutional transformer that integrates the characteristics of convolution with the ability of transformers to capture long-range dependencies, eliminating the need for any pretrained models. Building upon the Swin Transformer, Liu et al. (2023) devised a convolutional multi-head self-attention block. This design incorporates convolutional projection and window shift mechanisms, simultaneously offering local context and inductive bias. The deep fusion of convolution and transformer, leveraging the strengths of each, serves as inspiration for the design of our model.

## 2 Methods

### 2.1 Network architecture

**FIGURE 2:**
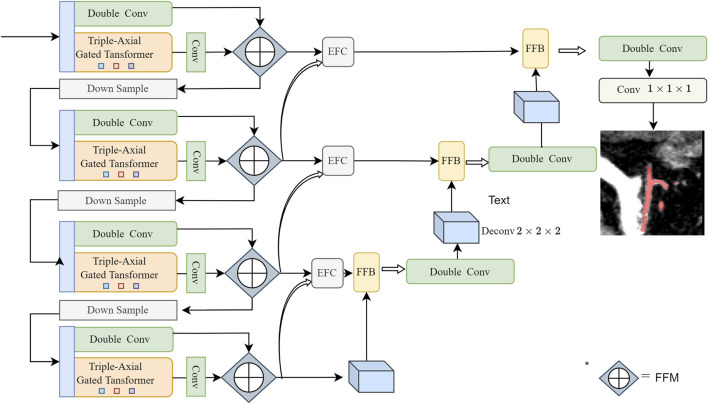
Overview of the structure of the proposed PE-Net: Two encoding layers consisting of CNN and TAGT, a skip connection layer consisting of EFC and FFB, and one decoding layer. The parallelly encoded features are fused using the Channel Attention-based method, as introduced in our previous work (Zhang et al., 2023)—a block named Feature Fusion Module (FFM). Before the operation, the features generated by TAGT are broadcasted to align their channel dimensions with the convolutional features.

Inspired by the great success of 3D Unet (Çiçek et al., 2016) and Swin Transformer (Liu et al., 2021), we propose a novel parallel network that combines transformer with CNN. The overall framework of our proposed network is illustrated in Figure 2. Our network consists of a contracting path (encoder), an expanding path (decoder), and skip connections.

Encoder: During the encoding process, we design two parallel branches. One branch follows the classic double-convolution encoder, while the other branch employs a transformer encoder based on axial attention. The features extracted from these two branches are fused in the channel dimension using the Feature Fusion Module (FFM), as introduced in our previous work.

Decoder: The decoder is responsible for progressively upsampling the extracted features from the encoder to the input image resolution. It consists of four layers, each composed of double convolutions, and upsampling is achieved through transpose convolutions.

Skip connections: The skip connection path comprises EFC and FFB. These modules respectively guide feature extraction from vessel boundaries and vessel regions to obtain more detailed vessel structure information.

At the network’s final layer, a 1 × 1 × 1 convolution followed by the softmax function is applied to generate segmentation probability maps. As an example, Table 1 shows the variations of parameters for each module in the first layer of the network, while further details for each module are described in the following Section.

**TABLE 1:** Details of the PE-Net architecture.

Layer 1	Input dimensions	Onput dimensions	Supplement
Double Conv1	1, 1, 128, 128, 128	1, 8, 128, 128, 128	K = 3, S = 1
TAGT1 head = 8 head_dim = 16	1, 128, 128, 1, 128	1, 1, 128, 128, 128	q, k, v = (131072, 1, 1, 16)
	1, 128, 128, 128, 128		q, k, v = (1024, 128, 128)
	1, 128, 128, 128, 128		q, k, v = (1024, 128, 128)
Conv	1, 1, 128, 128, 128	1, 8, 128, 128, 128	K = 3, S = 1
EFC	1, 1, 64, 64, 64	1, 8, 128, 128, 128	K = 2, S = 2

K denotes Kernel size, and S denotes stride size.

### 2.2 TAGT

**FIGURE 3:**
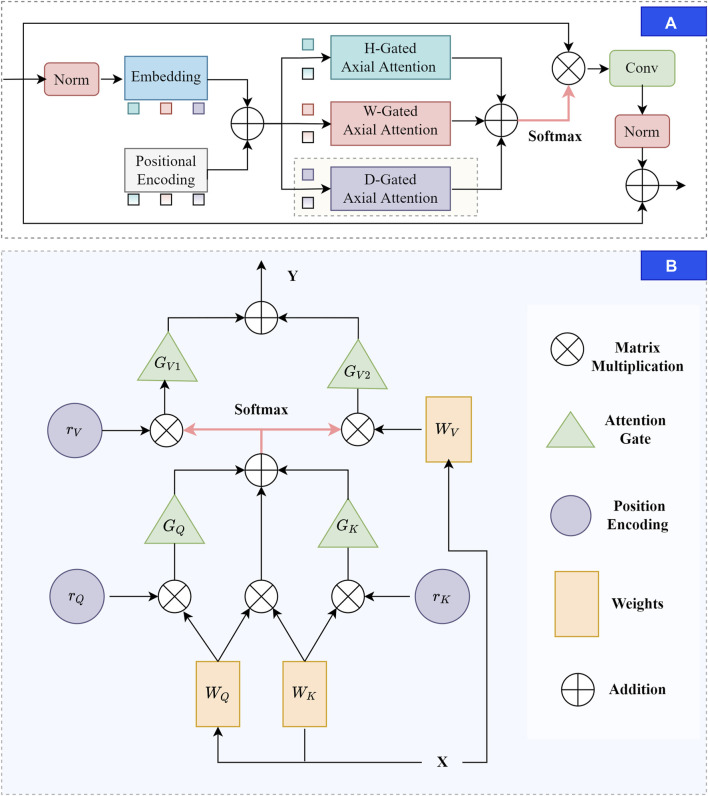
The diagram of the TAGT. **(A)** TAGT layer. Firstly, feature embedding and positional encoding are applied to the normalized features. Subsequently, corresponding features and position encoding are selectively chosen from the height, width, and depth axes for gated axial attention calculations. The results of the triple axial calculations are element-wise added, and the fused features undergo matrix multiplication with the original image to form the output. Convolution and normalization are applied, and a skip connection is added to stabilize training. **(B)** Three-dimensional gated axial attention layer, providing a detailed computational process for the shaded portion in **(A)**.

TAGT decomposes the transformer into three self-attention modules, breaking down the feature extraction process into three 1D operations along the depth, width, and height axes. It computes attention maps from these three operations and then combines them through summation and sigmoid to generate position weight maps. Building upon the conventional attention mechanism’s ability to consider query position deviations, Wang et al. (2020) advocates enhancing the model’s sensitivity to positional information by introducing relative positional bias terms for keys and values. Additionally, they attempt to perform attention calculations along the height and width axes, reducing parameter computation while ensuring the ability to capture long-range dependencies. Valanarasu et al. (2021) further introduces a gating mechanism for affinity calculations to further enhance the axial attention’s performance on small datasets. However, these efforts have been limited to 2D images. In this paper, we extend the axial attention to a 3D perspective by introducing computations along the depth direction. The overall structure of TAGT is illustrated in Figure 3. Below, we will provide a explanation of the derivation process of TAGT.

For a given input feature $X\in R^{C\times D\times W\times H}$ with channel *C*, depth *D*, height *H*, and width *W*. We first conducted data normalization to reduce the memory consumption of the model, accelerate model convergence, and improve training speed. Secondly, we used embedding layers to map features into vectors, including query vector *q*, key vector *k*, value vector*v*. We use matrices for batch computation, the definitions of the three matrices are described in Eqs 1–3:

$$v\left(X\right)=W^{v}X$$ (1)

$$q\left(X\right)=W^{q}X$$ (2)

$$k\left(X\right)=W^{k}X$$ (3)

where $v\left(X\right),q\left(X\right),k\left(X\right)\in R^{Z\times N}$ and *N* = *D* × *W* × *H*, *Z* is the embedding demention, *W* ^*v*^, *W* ^*q*^, *W* ^*k*^ are learnable parameters. In the conventional process of three-dimensional attention calculation, the output y of the self-attention layer can be described as $y_{i,j,t}=\sum_{d=1}^{D}\sum_{h=1}^{H}\sum_{w=1}^{W}\sigma \left(q_{i,j,t}^{T}k_{d,h,w}\right)v_{d,h,w}$ . Here, *q*_*i*,*j*,*t*_, *k*_*i*,*j*,*t*_, *v*_*i*,*j*,*t*_ denote query, key and value at any location, *i* ∈ {1, …, *D*}, *j* ∈ {1, …, *H*}, *t* ∈ {1, …, *W*}.

In TAGT, we transform the mapped vectors into four-dimensional features $R^{Z\times D\times W\times H}$ . Afterward, the transformed 4-dimensional features were added with corresponding positional encodings to form the ultimate input vector. This input vector underwent attention computations across the width, height, and depth dimensions. The attention update of a transformer with a triple-axis feature extraction module on the depth axis is shown in Eq. 4:

$$\begin{array}{ll} y_{i,j,t} & =\overset{D}{\underset{d=1}{\sum }}\sigma \left(q_{i,j,t}^{T}k_{d,j,t}+q_{i,j,t}^{T}r_{d,j,t}^{q}+k_{d,j,t}^{T}r_{d,j,t}^{k}\right)\\ & \;\times \left(v_{d,j,t}+r_{d,j,t}^{\nu }\right) \end{array}$$ (4)

where *σ* is function softmax *q*_*i*,*j*_, *k*_*i*,*j*_, *v*_*i*,*j*_, represents vector *q*, vector *k*, and vector*v* at any position, $r^{q},r^{k},r^{v}\in R^{D\times D}$ are relative position coding which are learnable.

We achieve the final extractor by adding attention gates to every item in Eq. 4 except the first item, the update on the depth axis can be described in Eq. 5.

$$\begin{array}{ll} y_{i,j,t} & =\overset{D}{\underset{d=1}{\sum }}\sigma \left(q_{i,j,t}^{T}k_{d,j,t}+G_{q}q_{i,j,t}^{T}r_{d,j,t}^{q}+G_{k}k_{d,j,t}^{T}r_{d,j,t}^{k}\right)\\ & \;\times \left(G_{v1}v_{d,j,t}+G_{v2}r_{d,j,t}^{\nu }\right) \end{array}$$ (5)

where the new added *G*_*q*_, *G*_*k*_, *G*_*v*1_, *G*_*v*2_ are all learnable parameters in network.

### 2.3 Vessel edge feature catcher

**FIGURE 4:**
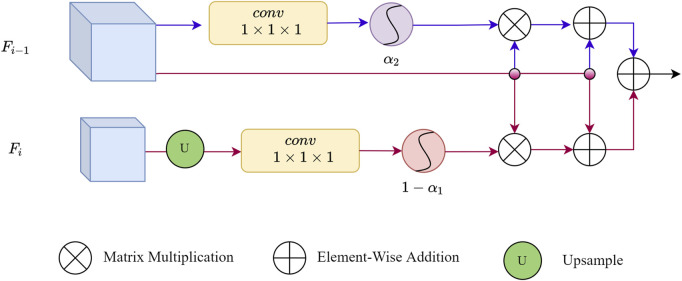
The architecture of EFC. The adjusted features in the encoding layer are denoted as *F*_*i*−1_ and *F*_*i*_, utilizing a double-layer path to ensure the extraction of edge features, distinguished by red and blue lines.

Continuous downsampling often leads to the loss of some details in the model, leading to the prediction of vascular edges often not being accurate enough. Xia et al. (2022) regards the intersection of the foreground and background of different layers as the target edge feature, and the edge weights of foreground vessels in layer (*i* − 1)^*th*^ can be obtained by subtracting the background probability map of layer *i*th from layer (*i* − 1)^*th*^. Hatamizadeh et al. (2020) employed a 1 × 1 × 1 convolution to design the edge gate module and verified its effectiveness in 3D medical image segmentation tasks. Inspired by the aforementioned two approaches, we designed our EFC which utilizes both the current layer itself and neighboring layers to ensure robust edge feature extraction. The detailed architecture of EFC is illustrated in Figure 4.

EFC discovers edge features through convolution and enhances them by increasing the voxel weights of the edge parts. Path 1 (red) first operates on features *F*_*i*_ generated in each encoding layer, $F_{i}\in R^{C\times D\times H\times W},i\in 2,3,4$ . We first increased the resolution of *F*_*i*_ to match that of *F*_*i*−1_. Then, we combined the features into a single channel using a 1 × 1 × 1 convolutional operation, passing them into ReLU to gain attention map *σ*_1_. Let *σ* be the function softmax, then processes can be represented using Eq. 6:

$$\sigma_{1}=\sigma \left(Re\left(C_{1}\left(Up\left(F_{i}\right)\right)\right)\right)$$ (6)

The weights captured in path1 can be described by Eq. 7:

$$A_{i-1}=1-\sigma_{1}\left(F_{i}\right)=1-\frac{1}{1+e^{-F_{i}}}$$ (7)

The corresponding edge feature can be captured by matrix multiplication, that is Eq. 8:

$$E_{i-1}=F_{i-1}*A_{i-1}$$ (8)

In the *i* − 1st layer, the final combined feature with the inclusion of edge features can be described using Eq. 9:

$$F_{sum_{i-1}^{1}}=F_{i-1}+E_{i-1}$$ (9)

In Path 2 (blue), the same operations as in Path 1 are performed on *F*_*i*−1_, except for the upsampling step, resulting in *σ*_2_ = *σ*(Re(*C*_1_(*F*_*i*−1_))). The final output containing edge features can be represented by Eq. 10:

$$F_{sum_{i-1}^{2}}=F_{i-1}*\sigma_{2}+F_{i-1}$$ (10)

The final blood vessel edge features generated by the dual pathways can be represented by Eq. 11:

$$F_{sum_{i-1}}=F_{sum_{i-1}^{1}}+F_{sum_{i-1}^{2}}$$ (11)

### 2.4 Deep feature fusion block

**FIGURE 5:**
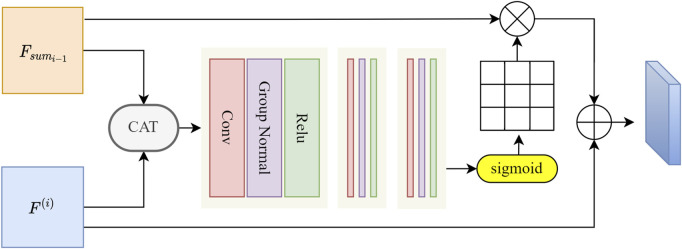
The illustration of the designed FFB. The two sets of features are initially summed along the channel dimension. Subsequently, three convolutional blocks are employed for feature selection, each convolutional block consists of a convolutional layer, a group normalization layer, and a ReLU layer. The corresponding convolutional layer sizes in the three blocks are 3 × 3 × 3, 3 × 3 × 3, and 1 × 1 × 1. Attention weights are computed and allocated using the sigmoid function.

Although EFC enhances blood vessel edge information, it also enhances some similar interfering vessels. To address this, we introduce FFB, which filters and retains prominent blood vessel features of IMA. We utilize attention mechanisms to generate refined attention features. Unlike the attention gate, the essence of FFB is a feature fusion module. Building upon the classic CAT operation in U-shaped networks, FFB introduces additional convolutions for feature selection. Therefore, we initially concatenate features along the channel dimension, followed by feature extraction and filtering. Specifically, as shown in Figure 5, we connect the final features $F_{sum_{i-1}}$ generated from the skip paths in each layer with the upsampled features *F* ^(*i*)^, $F^{\left(i-1\right)}=F_{sum_{i-1}}⊗Upsample\left(F^{\left(i\right)}\right)$ .

Subsequently, *F* ^(*i*−1)^ will be fed into three convolutional blocks, which selectively extractuseful vascular structure information from the fused features. This process can be expressed by Eq. 12:

$$W_{x-1}=g_{\mathrm{conv}}\left(F^{i-1};\theta \right)$$ (12)

where *g*_*conv*_ represents a set of three convolution operation functions. The variable *F* ^*i*−1^ refers to the integrated feature resulting from the combination of high-level and low-level features. Finally, the parameter *θ* is associated with the learning process involved in convolution.

In the end, we normalized *W*_*x*−1_ using the Sigmoid function and generated corresponding attention weights *A*_*x*−1_. This enables our network to learn to select more discriminative features, thereby achieving more accurate and reliable vascular region segmentation. Such improvements enhance the academic level of our algorithm.

## 3 Material, experiments and results

### 3.1 Dataset

**IMA dataset** is a self-made dataset that contains 60 anonymous patient upper abdominal vascular images from the Affiliated 2 Hospital of Nantong University in Nantong City, Jiangsu Province, China. The average size of the images was 512 × 512 × 90, with a classic voxel spacing of 1 × 1 × 1, each 3D IMA sample was cropped to 128 × 128 × 128. Three annotators and a professional expert were invited to annotate the vessels in the abdominal vascular images, including the background (label 0), IMA (label 1). The CT scans used in the experiment were obtained from a Siemens dual-source CT scanner (Somatom Force, Siemens Healthcare, Forchheim, Germany). The specific CT acquisition parameters were consistent with our previous work (Zhang et al., 2023).

**ASOCA dataset** consists of 60 coronary artery images, including 20 samples from patients with coronary heart disease and 20 normal samples, all of which are labeled. The remaining 20 unlabeled samples are used as the test set. These data were obtained from the Grand Challenge (https://asoca.grand-challenge.org/access/). The average scanning resolution is 200 × 512 × 512, but we resampled the dataset to a lower resolution, resulting in an average resolution of 200 × 256 × 256. The labeled dataset was split into training, testing, and validation sets in a ratio of 6:2:2.

### 3.2 Experimental setting

The platform used in this experiment comes from a deep learning computing platform with two NVIDIA RTX-3090 24 GB graphics cards. The operating system and version were Ubuntu 20.04, while the machine learning environment was configured with Torch 1.7.0 and CUDA 11.1. The program compilation environment was Python 3.6.12. During the training process, a 3-fold cross-validation was employed to partition the dataset. The Adam optimizer (Reddi et al., 2019) was used for network optimization, with an initial learning rate of 0.001 and a weight decay of 10^–8^. The learning rate was adjusted using CosineAnnealingWarmRestarts with eta_min set to 0.0001, and the total number of epochs was set to 600. The loss function used in this paper is presented in Eq. 13:

$$L_{PE-Net}=0.6\cdot L_{CE}+0.4\cdot L_{\mathrm{WCE}}+L_{\mathrm{DICE}}$$ (13)

### 3.3 Evaluation metrics

To comprehensively assess the segmentation performance of blood vessels and their edges, we utilized voxel-based metrics, including sensitivity (SEN), Dice Similarity Coefficient (DSC), Average Hausdorff Distance (AHD). SEN quantifies the proportion of true positive samples among all predicted results in the sample. A higher SEN corresponds to a higher proportion of true positive pixels, yet it neglects the ability to identify negative instances, rendering it unsuitable as a primary indicator for scoring segmentation performance. DSC utilizes the intersection of the predicted set and the ground truth to comprehensively evaluate sparse vessel segmentation in a large background context. A larger DSC indicates superior segmentation performance. AHD, calculated by measuring the closeness of corresponding points between the predicted and ground truth sets, offers a better assessment of edge accuracy in vessel segmentation. A smaller AHD reflects a smaller distance between the two sets, thus indicating better segmentation performance. DSC is sensitive to the internal filling of masks, while AHD is sensitive to the segmented boundaries. Considering vascular connectivity, we tend to favor models with smaller AHD. In cases where the difference in AHD is not substantial, higher DSC and SEN values also fall within the scope of consideration for the optimal model. The definition of evaluation metrics are illustrated in Eqs 14–16:

$$SEN=\frac{TP}{P}=\frac{TP}{TP+FN}$$ (14)

$$DSC=\frac{2|L\cap P|}{L\cup P}=\frac{2TP}{2TP+FN+FP}$$ (15)

$$\begin{array}{ll} AHD & =\frac{1}{2}\left(\frac{1}{P}\underset{p\in P,l\in L}{\max}\min d\left(p,l\right)\right.\left.+\;\frac{1}{L}\underset{l\in L,p\in P}{\max}\min d\left(p,l\right)\right) \end{array}$$ (16)

where sets *P* and *L* represent the predicted set and the label set, respectively, and their corresponding elements are denoted as *p* and *l*. TP, FN and FP represent the true positives, false negatives and false positives, respectively.

### 3.4 Experiment and results

#### 3.4.1 Backbone

Our network entails two parallel encoders. To validate the efficacy of the parallel architecture, we first compare the experimental data between 3D U-Net and the parallel structured 3D Unet+TAGT (CNN-TAGT). Subsequently, in order to determine the optimal convolutional structure within the convolutional branch, we individually assess the impact of substituting the convolutional branch with 3D ResUNet (Res-TAGT) and 3D DenseNet (Dense-TAGT) on segmentation outcomes. The quantified results of these experiments on the IMA dataset are presented in Table 2.

**TABLE 2:** Quantitative results of the backbone by 3 fold cross-validation for the IMA dataset (20 for trainning).

	3D Unet			CNN-TAGT			Res-TAGT			Dense-TAGT		
	SEN	DSC	AHD	SEN	DSC	AHD	SEN	DSC	AHD	SEN	DSC	AHD
Mean	0.8059	**0.8058**	6.8338	0.8612	0.8035	**6.1756**	0.8093	0.7478	7.4295	**0.9147**	0.6493	12.8518
Std	**0.0840**	0.0920	5.2341	0.0849	0.1658	**4.4352**	0.1017	0.1812	5.2341	0.1417	0.1875	5.7528
Med	0.8112	0.8320	6.5389	0.8986	0.8291	4.7947	0.8604	0.7909	6.5389	0.9510	0.7172	15.4803
Min	0.6554	0.5626	1.7071	0.7174	0.3284	1.2071	0.6274	0.2885	1.7071	0.6319	0.1182	2.6180
Max	1.0000	0.8919	16.4370	1.0000	0.9843	13.3660	0.9113	0.8881	16.4370	1.0000	0.7911	21.0060

We evaluate models using SEN, DSC and AHD. The best result is shown in bold text and the runner-up result is underlined.

We first conducted an analysis based on the average values, and further examined the data stability for results with insignificant differences in these averages. From Table 2, it is observed that the top two performers are predominantly the 3D Unet model and the CNN-TAGT model. In terms of AHD, CNN-TAGT achieved the best overall performance, outperforming 3D U-Net by a margin of 7.78%. Although Dense-TAGT exhibited superior sensitivity scores, its AHD was nearly double that of CNN-TAGT’s. This implies that Dense-TAGT possesses strong predictive capabilities for positive samples but also includes a substantial number of false positive results in its segmentation. While maintaining AHD performance, CNN-TAGT outperformed 3D Unet by 5.53% in SEN, with a value of 0.8059. Furthermore, the gap between the maximum and minimum values of SEN is relatively narrow, and the standard deviations are quite close, consistently staying within 0.9. This indicates that the advantage of CNN-TAGT over 3D U-Net is relatively stable. Due to the convolutional bias, the convolution-based 3D Unet model was unable to effectively capture global information and lacked efficient learning of positional information, leading to inevitable instances of mis-segmentation and reduced segmentation performance.

Regarding the DSC scores, the difference between the two models remained insignificant at 0.23%. Although the Res-TAGT model employed residual structures, its overall performance across various metrics was relatively mediocre. This could be attributed to the relatively shallow network architecture, which might not fully exploit the advantages of residual structures. In conclusion, CNN-TAGT exhibited superior and more stable experimental results compared to the other three networks. Through backbone comparative experiments, we determined that a simple double convolution structure is the best match with the transformer.

**FIGURE 6:**
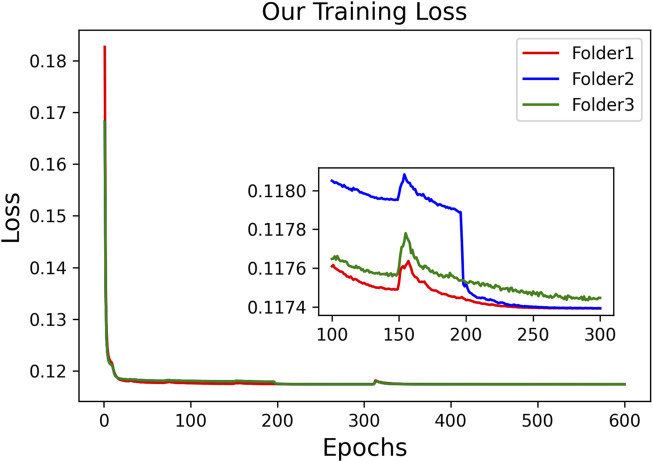
The training loss of CNN-TAGT during the 3-fold cross-validation.

Considering the poor performance of Res-TAGT and Dense-TAGT, we do not present their visualization results. The visualization results of 3D Unet and CNN-TAGT will be shown together in the subsequent ablation experiments. To further validate the stability of our model, we plotted the training curves of CNN-TAGT during the 3-fold cross-validation, as shown in Figure 6. We zoomed in on the curves between 100 and 300 epochs, and it can be observed that all three training runs achieved convergence around 250 epochs.

#### 3.4.2 Ablation studies

**FIGURE 7:**
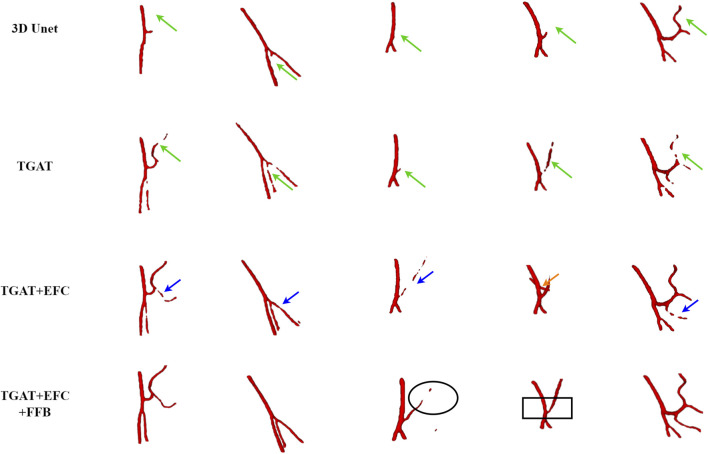
The visual results of the ablation experiments on the IMA dataset.

The PE-Net proposed in this study incorporates TAGT, EFC, and FFB modules. To confirm the effectiveness of these components, we conducted ablation experiments on each module. EFC and FFB were utilized to optimize the segmentation of small blood vessels, particularly enhancing the discriminative ability at vessel boundaries. Qualitative and quantitative results are presented in Figure 7 and Table 3, respectively. It is worth mentioning that the TAGT model represents the CNN-TAGT model that performed the best in the aforementioned backbone experiments.

**TABLE 3:** Quantitative results of PE-Net components on the IMA dataset, with the best results highlighted in bold.

Method	TAGT	EFC	FFB	Sen	DSC	AHD
Model 1	*✓*			0.8612	0.8035	6.1756
Model 2	*✓*	*✓*		**0.8779**	0.8109	6.3827
Model 3	*✓*	*✓*	*✓*	0.8774	**0.8137**	**5.9668**

As a supplement to the visualization results in the backbone experiments, we added a comparison with 3D Unet in the qualitative results. Observing the first column in Figure 7, we found that due to the inability to capture global information, 3D Unet had more vascular leakage problems. After introducing TAGT, the model detected more vascular details, as shown by the green arrows in the second column. However, due to the lack of precise capture ability for small vessels, the segmented vessels by TAGT appeared in a fragmented and uneven state. This situation was greatly alleviated after introducing the EFC module. The performance of EFC often demonstrated three characteristics: 1) Repairing the fractured vessels in TAGT by enhancing edge features, which can be observed through comparison with TAGT’s results; 2) Capturing more details of small blood vessels relative to TAGT, represented by the blue arrows; 3) Excessive learning of edge features resulted in the missegmentation of adjacent vessels, as shown in case 4 by the orange arrows. In the early models, these adjacent vessels were all classified as foreground vessels.

To address the above issues, we further introduced the FFB module, which accomplished feature selection through channel attention. FFB assigned significant weights to features related to target vascular structures, aiming to improve vascular segmentation and remove interference caused by EFC. The improved results are illustrated in the black boxes, where the vascular structures appeared brighter and smoother, indicating that the output features contained more representative information.

**FIGURE 8:**
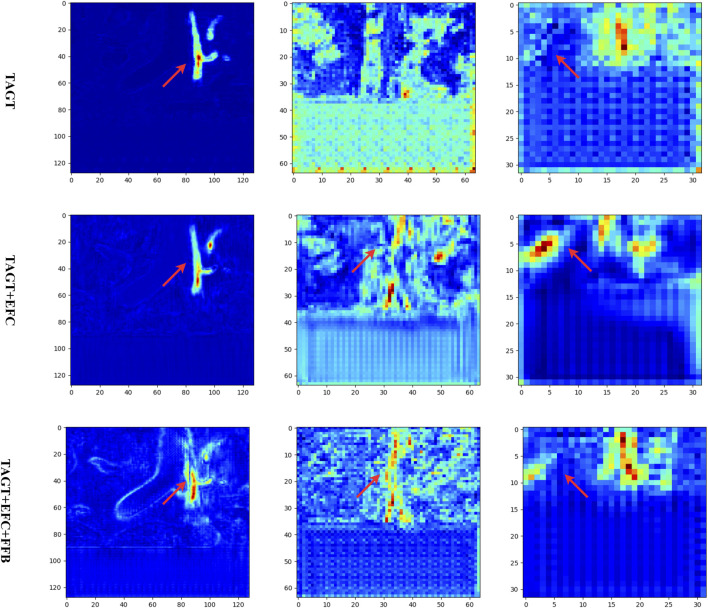
Feature maps of the ablation models. From left to right, the output feature maps of different models are shown at three encoding layers (with resolutions of 128, 64, and 32, respectively).

To further validate the effectiveness of each module, in Figure 8, we present the feature maps of various models in the contracting paths at resolutions of 128, 64, and 32. A random coronal slice from the volumetric data was selected, and the feature maps of all channels were averaged and projected. Comparing the first two rows, it was observed that the introduction of EFC resulted in wider blood vessels and more distinct features. The red regions spread both horizontally and vertically. Comparing the second and third rows, the red pixels became more compact, and the FFB module enhanced the connections between vessels while removing interfering vessels to some extent.

The quantitative results in Table 4 validated the above analyses. The comparison between model 1 and model 2 indicated that model2 with EFC achieved a 1.67% and 0.74% improvement in SEN and DSC scores, respectively, while AHD increased by 0.2, confirming the possibility of introducing neighboring vessel interference while capturing more true positive vessels. Comparing model 2 and model 3, the introduction of the FFB module resulted in a significant decrease in AHD, while maintaining almost unchanged SEN, and an increase of 0.28% in DSC score, suggesting that FFB employed a more reasonable fusion method for vascular features. FFB utilized channel attention to assign different weights to low-level and high-level features, selecting hidden features beneficial for vascular segmentation.

**TABLE 4:** Segmentation performance of different methods on IMA.

Dataset	Methods	SEN	DSC	AHD	Model parameter
2–6 IMA	3D Unet (Çiçek et al., 2016)	0.8059	0.8057	6.8338	21.54
	AU (Oktay et al., 2018)	0.8125	0.8082	8.2416	91.85
	CAS (Song et al., 2022)	0.8451	0.6052	21.0146	22.25
	MedT (Valanarasu et al., 2021)	0.8438	0.8136	12.2635	17.52
	Unetr (Hatamizadeh et al., 2022)	0.5694	0.5135	48.8125	88.38
	nnFormer (Zhou et al., 2021)	0.8441	**0.8227**	7.8673	143.26
	Ours	**0.8774**	0.8137	**5.9668**	58.42

#### 3.4.3 Comparison with state-of-the-art methods

**FIGURE 9:**
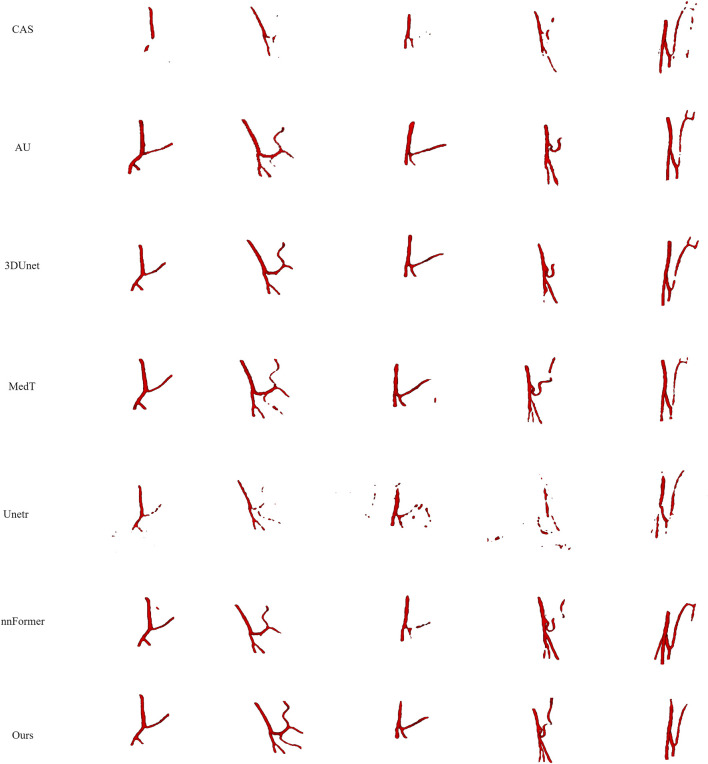
Visualization of segmentation results for different baseline models. From left to right, each column represents a case, and from top to bottom, they are CAS, AU, 3D Unet, MedT, Unetr, nnFormer, and our segmentation results, respectively.

In this part, we conducted experiments to validate the effectiveness of our proposed method using a self-made dataset. We compared our method with five different approaches, including the convolutional network 3D Unet, Attention Unet (AU) (Oktay et al., 2018), CAS(40), transformer-based method Unetr, MedT and nnFormer. The evaluation was performed on complete volume images rather than using patches to obtain the evaluation results. The qualitative and quantitative results are presented in Table 4 and Figure 9, respectively.

Firstly, we compared our model with convolutional models as shown in the first, second, and fifth rows of Figure 9. CAS achieved significant success by utilizing vessel region filtering and efficient feature fusion based on Dense blocks. However, we noticed that its performance on the IMA dataset was not optimistic, as it could not efficiently capture vascular features, even falling behind 3D Unet. CAS could only perform basic segmentation on the main IMA vessels and struggled with smaller branch vessels. We attribute this limitation to its Dense blocks, which contain only three 3 × 3 × 3 convolutions to reduce computational complexity. Consequently, this simple connection fails to fully exploit the advantages of the Dense structure, leading to a lack of capability in capturing complex vascular features. 3D Unet can roughly segment blood vessels’ overall structures, we observed that it missed some vessels in case 1–3, due to the limited receptive field of convolutions, which hindered its ability to explore branch vessels. Built upon the U-Net architecture, the AU introduces attention gates to improve target localization, emphasizing salient foreground features. Although AU demonstrates performance similar to U-Net overall, it does not enhance the continuity of vessels or deep vascular features. Attention gates are more effective in segmenting large organs, like the liver, but their efficacy is constrained in scenarios involving slender and discontinuous vessels, as seen in blood segmentation.

Next, we compared our model with transformer-based models. Unetr (the third row) performed poorly, as expected, as transformers usually require more extensive training data. Additionally, we noted that the original code’s epoch was set to 20,000, while we used only 600 epochs. Transformers introduce a large number of parameters, leading to higher time costs. Meanwhile, we also validated the transformer’s ability to model long-range features. Although Unetr struggled with vascular continuity, it could capture more vascular branches and achieve a preliminary representation of vascular morphology compared to convolutional networks. Assisted by the axial transformer, MedT effectively captures the complete branching of vessels. However, it is evident that within more intricate vascular patterns, the smoothness of blood vessels and the intensity of terminal vessels in MedT are notably inferior. In general, there is a lack of refinement capability for boundaries. nnFormer inherited the advantages of the nnUnet architecture and achieved satisfactory segmentation results. However, due to its limited capability in capturing edge features, we observed varying degrees of vascular discontinuity in cases 3 and 4. In case 3, our segmentation results surpassed nnFormer, while in case 5, nnFormer captured more extensive branch vessels.

Table 4 displays the parameter quantities and quantitative results of each model. It can be observed that our model achieves the best results in terms of AHD while having only half the parameter count of nnFormer. Additionally, our model demonstrates slightly higher or comparable values for SEN and DSC.

**FIGURE 10:**
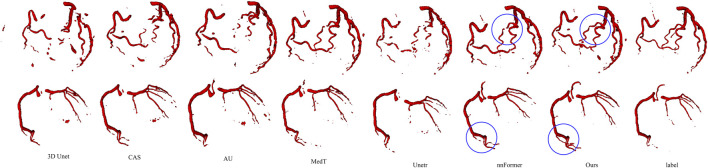
Visualization results of different methods on the ASOCA dataset.

Furthermore, we validated the performance of our model on ASOCA. The quantitative experimental results are presented in Table 5, showing that, compared to nnFormer, SEN achieves a similar score. Although it has a slight disadvantage in terms of DSC, our method obtained the best AHD. Visual results are displayed in Figure 10. The figure illustrates that the convolution-based models 3D U-Net, AU and CAS exhibit varying degrees of under-segmentation in tiny vessels, displaying extensive vascular disconnections. The Unetr performs similarly to its performance on the IMA dataset, failing to segment well-formed branching vascular structures. MedT yields rough vascular edges with a deficiency in refining boundaries, particularly showing a lower continuity in small vessels. In contrast, our model and nnFormer both manage to capture the overall vascular structure and our model particularly excels in learning vascular edge features, resulting in improved segmentation of small vessels with abrupt changes in direction, as highlighted by the blue circles.

**TABLE 5:** Segmentation performance of different methods on ASOCA.

Dataset	Methods	SEN	DSC	AHD
ASOCA	3D Unet (Çiçek et al., 2016)	0.7486	0.7214	19.4927
	AU (Oktay et al., 2018)	0.7501	0.7468	24.8731
	CAS (Song et al., 2022)	0.8205	0.8287	16.7368
	MedT (Valanarasu et al., 2021)	0.8146	0.8052	15.6824
	Unetr (Hatamizadeh et al., 2022)	0.7432	0.7025	26.3857
	nnFormer (Zhou et al., 2021)	**0.8373**	**0.8704**	7.7927
	Ours	0.8352	0.8137	**6.5873**

The corresponding experimental results demonstrate that our model possesses more advantages in terms of vascular continuity and branch integrity.

## 4 Summary and conclusion

### 4.1 Theoretical contributions

The prior knowledge of the three-dimensional structure of blood vessels has provided significant convenience for disease prevention, diagnosis, and treatment. In recent years, researchers have made significant contributions to the field of three-dimensional medical image processing, with a plethora of segmentation algorithms available for three-dimensional tumors and organs. However, the task of three-dimensional blood vessel segmentation remains highly challenging. Due to the difficulty in capturing features of small vessels and determining vessel edges, issues such as vessel leakage, missegmentation, and numerous vessel discontinuities often arise. The segmentation of 3D slender tubular blood vessels remains a topic worth researching together.

In this paper, we propose a new segmentation approach for three-dimensional segmentation of the inferior mesenteric artery in the abdomen. We designed a parallel architecture combining transformers and convolutions. We extended the gated axial attention mechanism to three dimensions and efficiently learned position deviations using the gating mechanism to enhance the network’s ability to capture features of small vessels, alleviating the transformer’s difficulty in learning image position encoding on small datasets. We designed the EFC to enhance the weight of edge voxels of blood vessels, thus boosting the learning of edge features and improving the continuity of blood vessels. FFB is used for selective feature fusion, retaining features significantly related to vascular structures to further optimize the results after EFC. On one hand, FFB performs deep connections on blood vessel structures further captured by EFC, and on the other hand, it assigns low weights to interfere vessels introduced by EFC, effectively removing them. The experimental outcomes indicate that, in contrast to state-of-the-art models, PE-Net achieves the best experimental results by simultaneously ensuring relatively high values for DSC and SEN, along with a better AHD. This underscores its performance with enhanced vascular continuity. Although our model has achieved good performance in the segmentation of IMA, in some vascular segmentation tasks, we had to lower the resolution to fit within the limited GPU memory. Due to the small size of blood vessel branches at the vascular periphery, directly reducing the resolution is not conducive to the overall segmentation performance of the vessels. nnUnet is an adaptive parameter model architecture capable of configuring parameters according to GPU memory. We are currently attempting to integrate PE-Net into the nnUnet architecture to ensure automatic configuration of tasks while preserving the full resolution.

### 4.2 Limitations and future research

Although our model has achieved good performance in the segmentation of IMA blood vessels, in some vascular segmentation tasks, we had to lower the resolution to fit within the limited GPU memory. Due to the small size of blood vessel branches at the vascular periphery, directly reducing the resolution is not conducive to the overall segmentation performance of the vessels. nnUnet is an adaptive parameter model architecture capable of configuring parameters according to GPU memory. We are currently attempting to integrate PE-Net into the nnUnet architecture to ensure automatic configuration of tasks while preserving the full resolution.

## Data Availability

The raw data supporting the conclusion of this article will be made available by the authors, without undue reservation.

## Abbreviations

IMA, inferior mesenteric artery; DSC, dice similarity coefficient; SEN, sensitivity; AHD, Average Hausdorff distance; CNNs, convolutional neural networks; TAGT, triple-axial gated transformer; EFC, edge feature capture; FFB, feature fusion block.
